# Significance of prohibitin domain family in tumorigenesis and its implication in cancer diagnosis and treatment

**DOI:** 10.1038/s41419-018-0661-3

**Published:** 2018-05-21

**Authors:** Jie Yang, Bin Li, Qing-Yu He

**Affiliations:** Key Laboratory of Functional Protein Research of Guangdong Higher Education Institutes, Institute of Life and Health Engineering, College of Life Science and Technology, Jinan University, 510632 Guangzhou, China

## Abstract

*Prohibitin* (*PHB*) was originally isolated and characterized as an anti-proliferative gene in rat liver. The evolutionarily conserved *PHB* gene encodes two human protein isoforms with molecular weights of ~33 kDa, PHB1 and PHB2. PHB1 and PHB2 belong to the prohibitin domain family, and both are widely distributed in different cellular compartments such as the mitochondria, nucleus, and cell membrane. Most studies have confirmed differential expression of PHB1 and PHB2 in cancers compared to corresponding normal tissues. Furthermore, studies verified that PHB1 and PHB2 are involved in the biological processes of tumorigenesis, including cancer cell proliferation, apoptosis, and metastasis. Two small molecule inhibitors, Rocaglamide (RocA) and fluorizoline, derived from medicinal plants, were demonstrated to interact directly with PHB1 and thus inhibit the interaction of PHB with Raf-1, impeding Raf-1/ERK signaling cascades and significantly suppressing cancer cell metastasis. In addition, a short peptide ERAP and a natural product xanthohumol were shown to target PHB2 directly and prohibit cancer progression in estrogen-dependent cancers. As more efficient biomarkers and targets are urgently needed for cancer diagnosis and treatment, here we summarize the functional role of prohibitin domain family proteins, focusing on PHB1 and PHB2 in tumorigenesis and cancer development, with the expectation that targeting the prohibitin domain family will offer more clues for cancer therapy.

## Facts

- PHB1 and PHB2 are widely distributed in cells and correlate with diverse diseases.
- PHB1 and PHB2 are involved in multiple biological processes in tumorigenesis like proliferation, metastasis and apoptosis.
- PHB1 and PHB2 are regulated by transcriptional regulation, post-transcriptional regulation and protein modification in cancer cells.
- Several small molecular inhibitors targeting PHB1 and PHB2 have significant impacts on cancer therapy.

## Questions

- Why PHB1 exerts controversial impacts on cell proliferation in different cancers?
- Which transcription factors regulate PHB1 expression in cancer cells?
- What are the mechanisms on regulation of PHB2 in cancer cells?
- Can PHB1 or PHB2 inhibitors enhance the treatment efficiency of chemotherapeutic drugs?

## Introduction

The *prohibitin* (*PHB*) gene was originally isolated and characterized as a candidate anti-proliferative gene in rat liver cells^1^, and the homologous human gene maps to chromosome 17q21-21^2–4^, encoding two ~33 KD proteins, PHB1 and PHB2^5^. PHB1 and PHB2 belong to the evolutionarily conserved band-7 family, or prohibitin domain family^5^. Both PHB1 and PHB2 are widely distributed in different cellular compartments including the mitochondria, nucleus and cell membrane, with diverse biological functions. PHB1 located in the inner mitochondrial membrane interacts with PHB2 to stabilize the mitochondria. As mitochondria are the main energy machines for biological activities in cells, expression changes in PHB1 or PHB2 in the mitochondria always induce mitochondria-related processes such as apoptosis^6, 7^.

PHB1 protein located in the cell membrane functions as viral or bacterial receptors to facilitate the entry of these microorganisms into host cells^8^. Meanwhile, PHB1 located in the lipid raft of the cell membrane interacts with and activates Raf-1, an evolutionarily conserved oncogene that activates ERK and promotes cancer development^9^. In addition, membrane-localized PHB2 also promotes cancer cell migration^10^. Moreover, PHB1 located in the nucleus binds to several transcription factors, such as p53, E2F, and pRb. Early studies claimed that PHB1 accumulates in the nucleus to induce cell cycle arrest and inhibit cell proliferation; however, this theory was challenged in recent years^5, 11, 12^. Nuclear PHB2 seems to be primarily involved in centromeric cohesion protection and promotion of cell growth^13^.

PHB1 and PHB2 are also involved in many biological processes related to tumorigenesis. Studies have found that overexpression of PHB1 and PHB2 results in cancer cell metastasis and apoptosis^10, 14–16^. Interestingly, although PHB1 was first identified as an anti-proliferative protein, the role of PHB1 in cancer cell proliferation remains controversial^17, 18^. As PHB1 and PHB2 have been shown to be differentially expressed in multiple cancers, PHB1 and PHB2 are potentially useful as new biomarkers and targets in cancer diagnosis and treatment.

Natural products provide an invaluable source of medicinal leads, presenting a significant impact on drug development. RocA is isolated from *Aglaia* species (Meliaceae), and fluorizoline is synthetized based on natural products from medicinal plants^19–21^. Both RocA and fluorizoline have been reported to interact with PHB1 directly and disrupt the interaction of PHB1 and Raf-1, therefore inhibits the activation of Raf-1/ERK signaling cascades and suppresss cancer cell growth and metastasis^14, 22^. RocA was also shown to significantly suppress cancer development in some drug-resistant cells^23^. Moreover, ERAP, a short synthetic peptide, and xanthohumol, a natural product from medical plants, were demonstrated to suppress cancer cell proliferation by targeting PHB2^24, 25^, indicating that drugs targeting PHB1 and PHB2 may be a promising strategy for cancer treatment.

Although there have been substantial advances in our understanding on the mechanisms of tumorigenesis, efficient remedies for diagnosis and treatment of cancer are still lacking. Considering the special localization and significant roles of prohibitin domain family proteins in cancer, the value of PHB1 and PHB2 in cancer treatment warrants further detailed study. Here, we summarize the current understanding on the functional role of PHB1 and PHB2 in biological processes, particularly tumorigenesis.

## Location and function of PHB1 and PHB2

The microenvironment in which proteins reside offers the perfect conditions to exert their function, therefore, localization has a large impact on protein function. According to the literature, both PHB1 and PHB2 are ubiquitously expressed, either in circulating form or in multiple cellular compartments, including the mitochondria, nucleus and plasma membrane^6, 11, 26, 27^.

### PHB1 and PHB2 locate in the inner mitochondrial membrane

PHB1 located in the inner mitochondrial membrane maintains mitochondrial stability by interacting with PHB2 to form a PHB1/PHB2 complex when mitochondria encounter metabolic stress^6, 28–30^. This process modulates the balance between mitochondrial fusion and fission events^31, 32^, thus maintaining a healthy mitochondrial network that protects cells from mitochondria-related apoptosis^7, 33, 34^. Former studies reported that loss of PHB1 and PHB2 in podocytes disrupts the activation of mTORC1 and inhibits kidney filtration^31, 35^. Levels of mitochondrial PHB1 are significantly decreased in the olfactory bulb, indicating that PHB1 is a driver of olfactory neurodegeneration in intermediate and advanced Alzheimer’s disease stages^36^. Another study demonstrated that loss of PHB2 from the mitochondrial membrane leads to tau hyperphosphorylation and neurodegeneration^37^.

Interestingly, experiments performed in transgenic mice illustrated that neuronal expression of mitochondrial PHB1 confers profound neuroprotection^38, 39^. A proteomics comparison between the substantia nigra (SN) and ventral tegmental area (VTA) dopaminergic neurons also demonstrated neuroprotection of mitochondrial PHB1 in Parkinson’s disease^40^. Moreover, PHB1 in the mitochondrial membrane is also involved in the regulation of sperm motility as shown by alterations in mitochondrial membrane potential in infertile men with poor sperm quality^41^. A recent study on PHB2 located in the inner mitochondrial membrane verified that PHB2 acts as a crucial mitophagy receptor involved in targeting mitochondria for autophagic degradation. Briefly, PHB2 was shown to bind the autophagosomal membrane-associated protein LC3 through an LC3-interacting region domain upon mitochondrial depolarization and proteasome-dependent outer membrane rupture, thus inducing eukaryotic mitophagy^42^.

### PHB1 and PHB2 locate in nucleus

Nuclear PHB1 modulates transcriptional activity directly through the interactions with various transcription factors, or indirectly through the interactions with chromatin remodeling proteins^5, 11, 12, 43^. The level of nuclear PHB1 can be down-regulated upon androgen treatment in cancer cells, indicating that PHB1 has a regulatory role in cell cycle progression^44^. In prostate cancer cells, PHB1 interacts with and suppresses E2F1 expression, repressing E2F-mediated transcription and inducing cell cycle arrest^41, 45^. PHB1 in the nucleus also functions as a potent transcriptional corepressor for estrogen receptor α (ERα) to abrogate cell proliferation^46^. Moreover, the investigation on paclitaxel resistance in cancer cells demonstrated that ERα promotes PHB1 mitochondrial-to-nuclear translocation to regulate estrogen-dependent paclitaxel resistance^47^.

In leukemic cells, PHB1 is strongly expressed in the nucleus and is a useful biomarker for the identification of leukemia subtypes^48^. PHB2 in the nucleus is phosphorylated by AKT at Ser-91, acts as a putative nuclear substrate of AKT and induces the differentiation of acute promyelocytic leukemia cells^49, 50^. Other studies demonstrated that PHB2 is essential for protecting centromeric cohesion in the regulation of sister-chromatid cohesion during mitosis, indicating that PHB2 is necessary for proper mitotic progression^13^.

### PHB1 and PHB2 locate in cell membrane

Some PHB1 proteins have been reported to interact with low density detergent-insoluble lipid raft domains in the plasma membrane^26, 51^, acting as transmembrane adapters to activate downstream signals^14, 52, 53^. Previous studies also identified that PHB1 on plasma membrane acts as a viral receptor protein to facilitate virus entry into host cells^8, 54, 55^. In addition, studies found that expression level of PHB1 on T cell surfaces is significantly up-regulated when T cells are activated^56^. Interestingly, another study claimed that interaction of PHB1 and Vi capsular polysaccharide (Vi) on T cell plasma membrane is crucial to inhibit T cell activation^57, 58^. Moreover, PHB1 located on *S. typhi*-host cell plasma membrane interacts with Vi to down-regulate early inflammatory responses, indicating that PHB1 contributes to the pathogenesis of typhoid fever^58^. In pancreatic cancer tissue, the expression level of PHB1 on plasma membrane was found to be higher than that in normal tissues, indicating an important role of PHB in tumorigenesis^59^. In recent studies, PHB1 located on the platelet membrane was demonstrated to be involved in PAR1-mediated human platelet aggregation^60, 61^, while PHB2 located in rhabdomyosarcoma (RMS) cell membrane was reported to act as a regulator in IGFBP-6-induced RMS cell migration through its interaction with insulin-like growth factor (IGF)-binding protein (IGFBP)-6^10^.

**Fig. 1:**
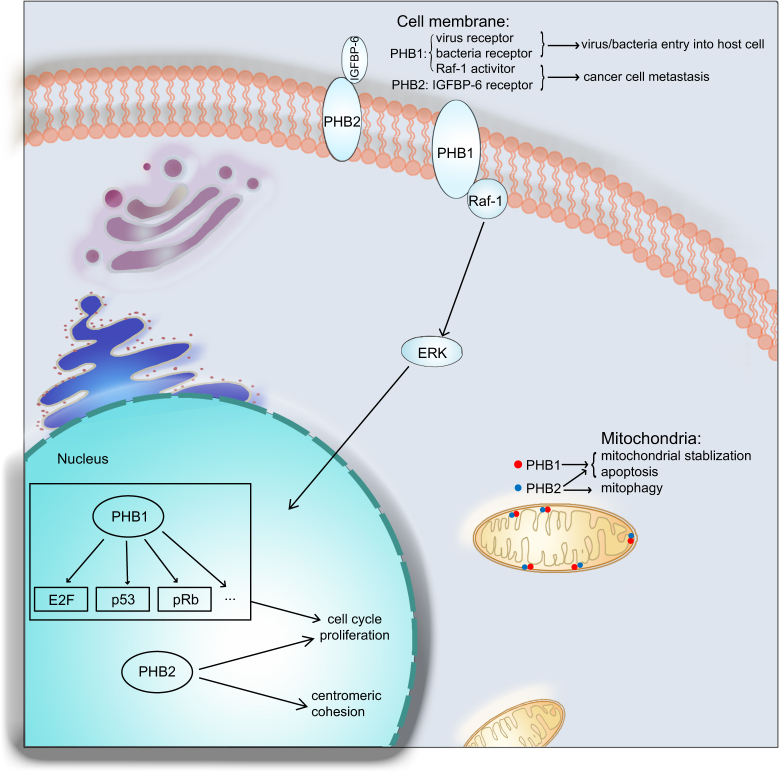
Location and function of PHB1 and PHB2. PHB1 and PHB2 locate mainly in mitochondria inner membrane, nucleus and cell membrane. In mitochondria: PHB1 and PHB2 maintain mitochondrial stabilization and cell survival, and PHB2 also functions as a regulator in mitophagy. In nucleus: PHB1 binds to some transcription factors and play an important role in cell cycle process and cell proliferation, and PHB2 protects the centromeric cohesion during mitosis. In cell membrane: PHB1 not only acts as virus receptor to facilitate virus entry into host cells, but also interacts with Raf-1 and activates Raf-1/ERK signaling cascades to promote cancer cell metastasis, whereas PHB2 promotes cancer cell metastasis mainly through the interaction with IGFBP-6

Collectively, PHB1 and PHB2 located in different cellular compartments function in different biological processes, which are involved in multiple diseases (Fig. 1). The functional role of PHB1 and PHB2 in tumorigenesis and the mechanism involved requires further investigation for potential implication of prohibitin domain family proteins in tumor diagnosis and treatment.

## Role of PHB1 and PHB2 in tumorigenesis

**Fig. 2:**
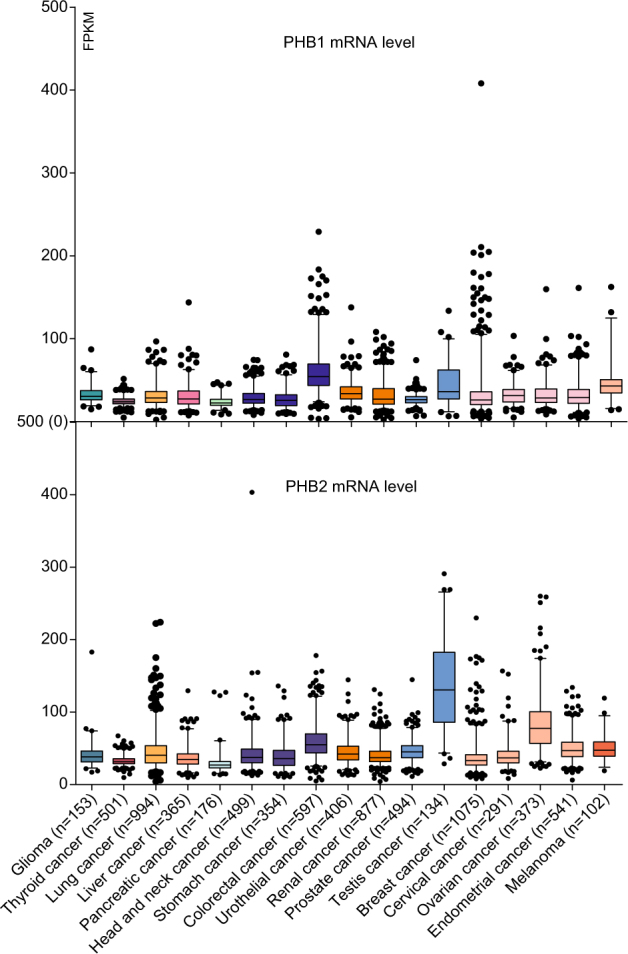
The mRNA expression levels of PHB1 and PHB2 in multiple cancers. RNA sequencing data were downloaded from The Cancer Genome Atlas (cancergenome.nih.gov). Up: PHB1 mRNA level; down, PHB2 mRNA level. Cancer number = 17; Whiskers: 2.5–97.5 percentile; FPKM Fragments Per Kilobase of exon model per Million mapped fragments

**Fig. 3:**
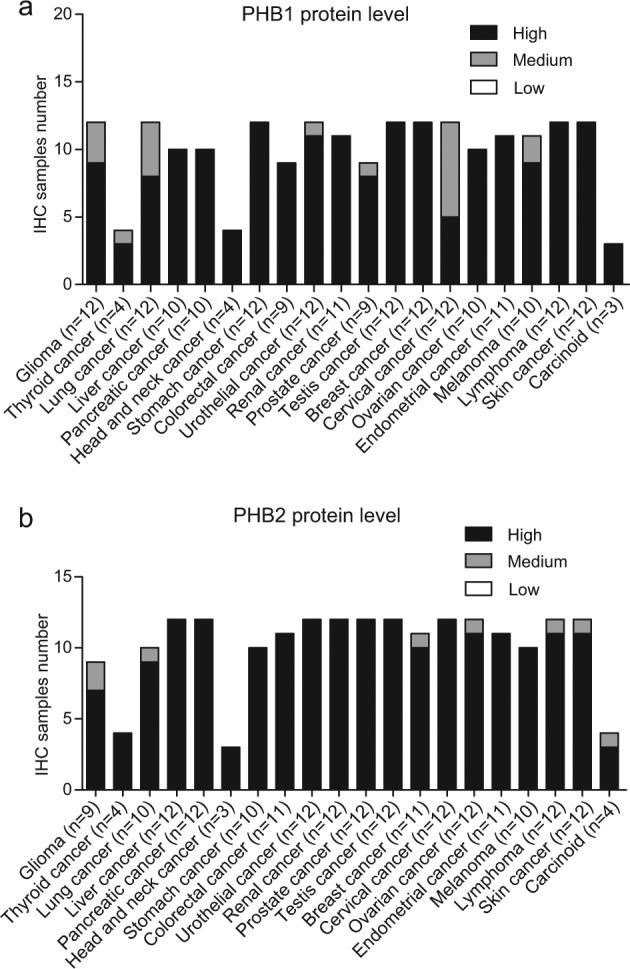
The protein expression levels of PHB1 and PHB2 in multiple cancers. The immunohistochemistry data of clinical samples were downloaded from The Human Protein Atlas (www.proteinatlas.org). **a** PHB1 protein level. **b** PHB2 protein level. Cancer number = 20. High, staining score >75%; Medium, 75% > staining score > 25%; Low, staining score < 25%

Data from The Cancer Genome Atlas (TCGA) and The Human Protein Atlas show that PHB1 and PHB2 are widely expressed in diverse cancers, at both mRNA and protein levels (Fig. 2, Fig. 3)^62^. A lot of evidence demonstrated that PHB1 and PHB2 are involved in biological processes of cancer development, such as proliferation, apoptosis, and metastasis.

### Roles of PHB1 and PHB2 in cancer cell proliferation

Since the original identification of PHB1 as a proliferation suppressor in rat liver cells^1^, the effect of PHB1 on cancer proliferation has been studied. Some studies found that PHB1 inhibits breast cancer cell proliferation by up-regulating androgen receptor (AR) expression in estrogen receptor (ER)-positive breast cancer cells^15, 63^. PHB1 also inhibits proliferation of human osteosarcoma MG-63 cells by interacting with tumor suppressors such as p53^64^. In glioma cells, PHB1 expression is down regulated by miR-26a, which interferes with the regulation of PHB1 on expression levels of HIF-1 and VEGF, as well as tumor growth^65, 66^. Moreover, PHB1 is significantly down regulated in nasopharyngeal carcinoma and hepatocellular carcinoma^17, 67^, indicating that PHB1 may be a promising diagnostic biomarker in these cancers.

However, PHB1 exhibits opposing functions in other cancer types. For example, down-regulation of PHB1 inhibits cancer cell proliferation by inducing G1-G0 arrest in esophageal squamous cell carcinoma (ESCC)^18^. Overexpression of PHB1 was verified in gallbladder cancer tissues, and further studies identified that PHB1 promotes cancer cell proliferation by activating ERK and downstream signaling pathways^68^. Similarly, PHB1 was also demonstrated to be overexpressed in bladder cancer tissues; mechanistically, AKT phosphorylates PHB1 at T258, inducing mitochondrial localization of PHB1 and leading to cancer cell proliferation^69, 70^. Moreover, PHB1 is overexpressed in ovarian epithelial tumors and induces cell proliferation^71, 72^. In estrogen-dependent cancers such as breast cancer, investigators reported that BIG3, a key molecular regulator in the ER signaling pathway, inhibits the nuclear transportation of the PHB2/REA transcription complex, abolishing the inhibitory effect of the PHB2/REA complex on ERα transcriptional activity^73, 74^. PHB2 was also shown to promote hepatocellular carcinoma growth and malignancy progression in the hypoxic tumor microenvironment^75^.

### Roles of PHB1 and PHB2 in cancer cell metastasis

Despite many advances in the diagnosis and treatment of cancer, tumor metastasis remains largely incurable and up to 90% of cancer-related deaths are caused by metastatic disease rather than primary tumors^76–78^. Therefore, efficient biomarkers or targets involved in cancer metastasis are urgently needed to be identified. Interestingly, unlike its controversial role in cancer cell proliferation, PHB1 expression levels are significantly correlated with tumor metastasis and poor prognosis according to the current literature. In many cancers, the Raf-ERK signaling pathway is constitutively activated, promoting cancer cell metastasis. Recent studies have confirmed that PHB1 in lipid rafts is crucial for the activation of Raf-1 and downstream signals. For example, studies in HeLa and CL1-0 cells showed that phosphorylation of PHB1 at T258 and Y259 is necessary for the activation of Raf-ERK signaling cascades supporting cancer cell metastasis^14, 79^. A similar mechanism was observed in pancreatic ductal adenocarcinoma and gallbladder cancer as well^22, 68^.

Some researches claimed that a polarized distribution of PHB1 in cells controls the migration direction of colorectal cancer cells. PHB1 can relocate to the luminal side of cells, face extracellular VEGF and indicate the direction of colorectal cancer cell invasion^80^. In lung cancer cells, studies verified that association of phospho-PHB1 T258 with MEKK1 activates the Snail, the repressor of E-cadherin, enhancing epithelial-mesenchymal transition (EMT) and lung cancer migration/invasion^81, 82^. PHB1 overexpression is also reported in invasive breast carcinoma, indicating that PHB1 is a potential biomarker in breast cancer^83, 84^. In prostate cancer, PHB2 was reported to interact with AKT2 and negatively regulate AKT2 expression, inducing cancer cell migration and malignancy^49^.

### Roles of PHB1 and PHB2 in cancer cell apoptosis

Apoptosis is defined as programmed cell death that plays a fundamental role in organism development and tissue homeostasis. Disruption of apoptotic signaling results in multiple diseases^85–87^. Apoptosis is also important for cancers to eliminate damaged cells that cannot be repaired, maintaining cancer cell vibrancy and aggressiveness^88^. On the other hand, induction of apoptosis is a promising strategy for cancer treatment to eliminate cancer cells^89–91^. The role of PHB1 in cancer cell apoptosis has been explored in various studies. In breast and colon cancers, researchers demonstrated that PHB1 binds to the p53 induced gene 3 (*PIG3*) promoter motif (TGYCC) _15_ directly, promoting PIG3-mediated, p53-dependent cancer cell apoptosis^92, 93^. Another study reported that cholesterol insufficiency in prostate cancer cells causes up-regulation of PHB1, inducing cell cycle arrest and apoptosis^94^. Furthermore, a series of experiments in colon cancer cells and melanoma cells confirmed that the direct interaction of PHB1 with trifluorothiazoline is necessary for trifluorothiazoline-induced apoptosis^19, 32^. This phenomenon was also observed in abrin-triggered apoptosis^95^ and the retinoic acid-resistant leukemia cell line NB4-R1^96^. Interestingly, PHB1 expression significantly decreases, with its localization shifting from cytoplasmic to nuclear, and the co-localization of PHB1 with tumor suppressors such as p53 and Rb promotes apoptosis in cholangiocarcinoma^97^. Moreover, PHB2 can translocate from the mitochondria to the nucleus during capsaicin-induced apoptosis^16^. In ER-positive breast cancer, overexpression of PHB1 in MCF-7 and T47D cells significantly induces cell apoptosis^15^, and the same phenomenon was detected in both gastric and liver cancer cells^98, 99^.

In fact, PHB1 and PHB2 not only function in biological processes like cancer cell proliferation, metastasis and apoptosis in tumorigenesis and cancer development, but also play a role in modulating other processes such as cell differentiation in hepatocarcinoma, neuroblastoma and acute promyelocytic leukemia^50, 100, 101^ and mitophagy^42^. As the functional role of PHB1 and PHB2 in cancer becomes increasingly well defined, it is necessary to evaluate the upstream regulatory mechanisms of PHB1 and PHB2 in tumorigenesis to facilitate our understanding on the prohibitin domain family proteins and to generate clues for targeted drug development.

## Regulation of PHB1 and PHB2 in cancers

Proteins in cells are usually regulated in three ways: transcriptional regulation^102, 103^, post-transcriptional regulation at the mRNA level^104, 105^ and protein modification^106^ including degradation^107^ and structural regulation^108^. Transcriptional regulation mainly involves the regulation by transcription factors and epigenetic mechanisms^109, 110^, while post-transcriptional regulation involves RNA interference^111, 112^. Based on recent studies, all the three approaches of regulation seem to be involved in controlling prohibitin domain family proteins’ effects on cancer cells.

### PHB1 is regulated at the transcriptional level

At transcriptional level, researchers found that PHB1 expression level is associated with PHB1 genome copy number and a 3′ untranslated region (UTR) 1630 C > T polymorphism in gastric cancer^113^. Interestingly, the PHB1 genotype C1703T in the 3′-UTR is correlated with an increase in the risk for melanoma in a high ultraviolet radiation region in Brazil^114^. The expression of PHB1 was found to significantly increase in the thyroid tumor cells treated with trichostatin A (TSA) and sodium butyrate (NaB), two histone deacetylase inhibitors, demonstrating that HDAC1/2 regulates both PHB1 transcription and alternative splicing^115^. Unfortunately, it remains unclear which transcription factors are responsible for PHB1 transcription.

### PHB1 and PHB2 are regulated at the post-transcriptional level

At the post-transcriptional level, miR-27a targets the 3′-UTR of the *PHB1* gene and down-regulates its expression in prostate cancer^116^. Likewise, the same mechanism has been confirmed in gastric adenocarcinoma cells and glioma cells^117, 118^. Interestingly, miR-26a also binds directly to the 3′-UTR of *PHB1*, inhibiting its expression in glioma cells^66^. In human melanoma cells, studies showed that miR-195 binds to the 3′-UTR of *PHB1* as well^119^. Researchers also demonstrated that miR-539 binds *PHB2* and suppresses its expression to induce mitochondrial fission and apoptosis^120^. Moreover, one study recently confirmed that a long noncoding RNA (lncRNA), prohibitin gene pseudogene 1 (*PHBP1*), which shares the high level of the nucleotide sequence identity (91.3%) with its cognate gene *PHB1*, promotes the stabilization of *PHB1* mRNA by forming a *PHBP1 RNA*/*PHB1 mRNA* heteroduplex in complementary regions, increasing the expression of PHB1^18^.

### PHB1 is modified by AKT

Protein modifications such as phosphorylation, glycosylation, ubiquitylation and small ubiquitin-related modifier (SUMO) determine the maturity, stability and activity of proteins^121–123^. For PHB1, phosphorylation or dephosphorylation at different amino acid sites results in different effects on tumorigenesis. Recent studies have focused on the phosphorylation of PHB1 at Tyr114, Ser121, Thr258, and Tyr 259^124^, with Thr258 and Tyr 259 phosphorylation well characterized in cancer cells. Phosphorylation of PHB1 at Thr258 in lipid rafts is necessary for the interaction of PHB1 with Raf-1. AKT phosphorylates PHB1 at Thr258, inducing the activation of Raf-1 and the Raf-1/ERK signaling pathway, resulting in cancer metastasis^14, 79^. Interestingly, Tyr259 phosphorylation induces the activation of Thr258 and the downstream Raf-1/ERK signaling cascades.

**Fig. 4:**
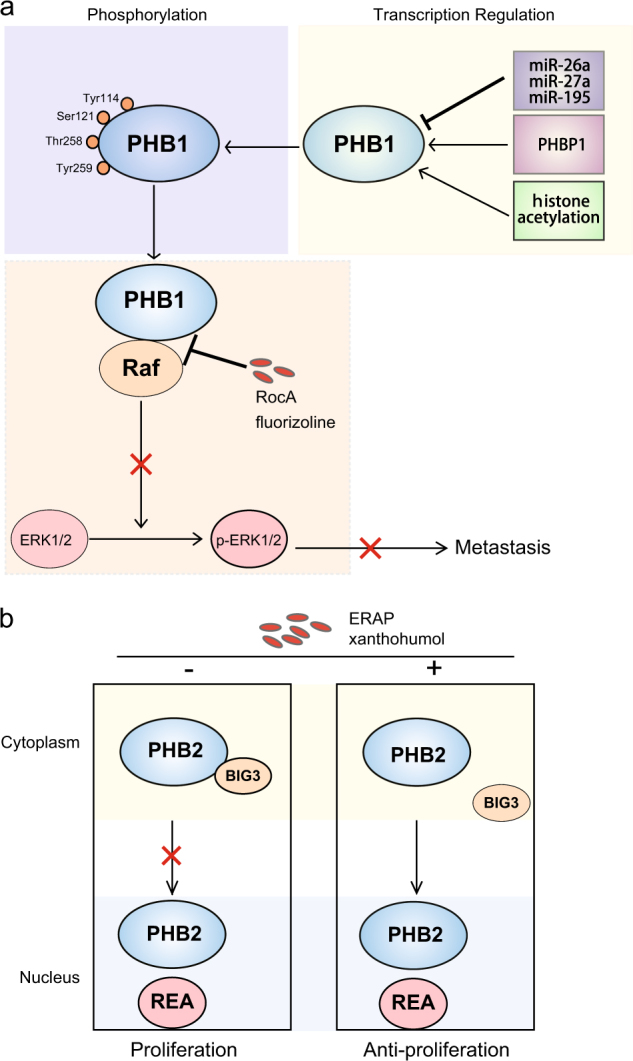
Regulation of PHB1 and PHB2 in cancer. **a** PHB1 can be down-regulated by miR-26a, miR-27a, and miR-195; the lncRNA named *PHBP1* directly binds to and maintain the stabilization of PHB1 mRNA; the acetylation of histone H3 can also increase the expression of PHB1; phosphorylation of PHB1 at different amino acids determines the activation and function of PHB1. Small inhibitors including RcoA and fluorizoline directly bind to PHB1 and interrupt its interaction with Raf, thus blocks Raf-ERK signaling cascades and suppress cancer metastasis. **b** The short peptide, ERAP, and the natural product derived from medical plants, xanthohumol, could disrupt the PHB2/BIG3 interaction directly and lead to the translocation of PHB2 from cytoplasm to nucleus, thereby, induce cell growth arrest in some estrogen-dependent cancers

Collectively, transcription of PHB1 is regulated by histone acetylation, microRNAs, and some lncRNAs. Moreover, phosphorylation of PHB1 at Thr258 and Tyr259 induced by AKT is important for cancer metastasis (Fig. 4a). Unfortunately, data on the upstream regulation of PHB2 is still lacking. Understanding of the regulation of prohibitin domain family and the related biological processes will offer us clues for further investigations on cancer diagnosis and treatment.

## Value of PHB1 and PHB2 as therapeutic targets in cancer treatment

Many efficient targeted drugs have been developed for cancer treatment, balanced by increasing instances of clinical drug resistance^125, 126^. Mechanistic studies found that most target genes or protein mutants develop resistance to targeted drugs over a period of time^127–129^. Thus, new effective targets are always needed. As summarized above, PHB1 acts as a tumor promoter in most cancer types, and thus is a potential target for novel drug development, especially the PHB1 located in the cell membrane. In fact, several small inhibitors targeting PHB1 or PHB1/Raf-1 or PHB2 interactions have been identified, and tumors were significantly inhibited by these drugs.

### Drugs targeting PHB1

Rocaglamide (RocA), a naturally occurring compound isolated from medicinal plants belonging to the genus *Aglaia* family *Meliaceae*^20^, was used for treatment of coughs, injuries, asthma, and inflammatory skin diseases. RocA directly targets PHB1 and prevents its interaction with Raf-1, resulting in impaired ERK1/2 activation in leukemic cells^130^. The effect of RocA was further displayed in pancreatic ductal adenocarcinoma, where RocA inhibits the interaction of PHB1 and Raf-1, significantly suppressing cancer cell growth and metastasis^22^. Interestingly, RocA can reverse Raf-1-dependent resistance to vemurafenib, inhibite cell growth and induce apoptosis in melanoma cells^23^, indicating that RocA has potential value for use in vemurafenib-resistant cancers.

Recently, researchers demonstrated that a small molecular inhibitor fluorizoline, synthetized based on natural products from medical plants, also prevents Ras-Raf interaction in lung cancer cell and inhibits tumor growth and metastasis^131^. Moreover, fluorizoline induces mitochondrial-dependent apoptosis by targeting at PHB1 in a p53-independent manner in MEF cells, as well as in multiple cancers^19, 33, 132^. The combined use of RocA and fluorizoline may be a promising strategy for cancer treatment (Fig. 4a).

### Drugs targeting PHB2

As PHB2 is involved in estrogen-dependent cancers such as breast cancer, and BIG3 promotes cancer cell proliferation by inhibiting PHB2 nuclear translocation and promoting the activity of ERα, the BIG3/PHB2 interaction is necessary for tumorigenesis^73, 74^. Therefore, inhibition of the interaction of BIG3-PHB2 is a feasible strategy in estrogen-dependent cancer therapy. In fact, researchers isolated the stable ERα activity-regulator synthetic peptide (ERAP: 165-177 amino acids), a short peptide derived from α-helical BIG3 sequence that specifically binds to PHB2 and competitively prevents the BIG3/PHB2 interaction, and then showed that ERAP promotes PHB2/REA complex nuclear translocation and suppresses cancer cell proliferation in vitro and in vivo^133^. Moreover, PHB2 released from the BIG3-PHB2 complex by ERAP treatment reduces phosphorylation levels of AKT and MAPK, resulting in significant suppression of proliferation in ERα-positive breast cancer cells. More importantly, the tumor suppressive effects of ERAP are enhanced by combined use with tamoxifen, indicating that ERAP treatment can reverse tamoxifen resistance and enhance tamoxifen responsiveness in ERα-positive breast cancer cells^24, 25^. The same research team also identified a natural product derived from medical plants named xanthohumol that could interrupt BIG3/PHB2 interaction and suppress ERα-positive breast cancer cell proliferation (Fig. 4b)^134^.

New target exploration is a continuous effort for new drug development, offering more opportunities to overcome drug resistance in cancer treatment. Based on the special localization and clear mechanism of PHB1 in tumorigenesis, there is a bright future for developing high-efficiency drugs to target PHB1 for cancer treatment. PHB2 inhibitors will also play an important role in estrogen-dependent cancer treatment, especially in premenopausal women.

## Conclusion and future perspectives

Since the *PHB* gene was first isolated and characterized in rat liver in 1989, researchers have been studying this “anti-proliferation” gene to elucidate its role in biological process and diseases.

Biomarkers and genes for targeted drug development are important for cancer diagnosis and treatment, and drugs developed based on proteins such as EGFR have been extensively used for clinical treatment^135^. However, increasing numbers of patients are presenting with drug resistance when treated with extant drugs for long periods of time^128^. Thus, exploration of new targets is urgently needed. Differential PHB1 expression has been identified in multiple cancers as compared with normal tissues; hence, the PHB1 protein, especially when located in the cell membrane, may be a perfect choice for targeted drug development, as drugs could target PHB1 directly without transportation into cells. The observations that small molecule inhibitors RocA and fluorizoline target PHB1, and a short peptide ERAP and xanthohumol target PHB2, provide us stimulation in anti-cancer drug design. At least, modifying these existing drugs to increase their efficiency, or screening more potent inhibitors targeting PHB1 and PHB2 may lead to breakthroughs in cancer therapy.
